# Improved outcomes after anterior vertebral tethering for AIS using ideal versus acceptable indications

**DOI:** 10.1007/s43390-026-01324-y

**Published:** 2026-03-19

**Authors:** John T. Braun, Sofia C. Federico, David F. Lawlor, Daniel P. Croitoru, Brian E. Grottkau

**Affiliations:** 1 Massachusetts General Hospital, 55 Fruit St., Yawkey 3E, Boston, MA 02114 USA; 2 Massachusetts General Hospital, Boston, MA USA; 3 Massachusetts General Hospital, Harvard Medical School, Boston, MA USA; 4 Dartmouth Hitchcock Medical Center, Geisel School of Medicine at Dartmouth, Lebanon, NH USA

**Keywords:** Scoliosis, Anterior vertebral tethering, Ideal indications

## Abstract

**Introduction:**

Though anterior vertebral tethering (AVT) has been proposed as an alternative to fusion surgery for AIS, ideal versus acceptable indications for this novel procedure have yet to be established. This study compared outcomes after AVT in AIS patients with ideal indications versus those with acceptable indications and 1, 2, or 3 risk factors.

**Methods:**

One hundred eighty-five consecutive AIS patients were treated with AVT for thoracic and thoracolumbar curves 33–72° with 140 patients having 2–11 year follow-up data. Four groups were analyzed: 1 group with ideal indications and no risk factors and 3 groups with acceptable indications and 1, 2, or 3 risk factors. Ideal indications included curve magnitude of 40–60°, curve flexibility ≥ 50%, and skeletal maturity graded at Risser 0–2 and/or Sanders 3–5. Those patients with acceptable indications were considered to have 1, 2, or 3 risk factors if they failed to meet 1, 2, or 3 of these criteria. Radiographic outcomes were graded as excellent ≤ 25°, good 26–39° fair ≥40°, or poor ≥ 50° and/or requiring fusion.

**Results:**

One hundred forty AIS patients (118F/22 M) treated with AVT had 2–11 year follow-up. Patients with ideal indications (*n* = 42) demonstrated 95% good or excellent outcomes with a 27% overall tether rupture rate and 2% revision surgery rate. Patients with acceptable indications and 1 risk factor (*n* = 72) had 86% good or excellent outcomes with a 36% overall tether rupture rate and 15% revision surgery rate. Those with 2 risk factors (*n* = 21) had 67% good or excellent outcomes with a 33% overall tether rupture rate and 19% revision surgery rate. And those with 3 risk factors (*n* = 5) had 40% good or excellent outcomes with a 40% overall tether rupture rate and 40% revision surgery.

**Conclusion:**

Outcomes after AVT for AIS were better in patients with ideal versus acceptable indications. Though the chance of a good or excellent outcome at final follow-up was best in patients with ideal indications (95%), this dropped steadily with the accumulation of 1 (86%), 2 (67%), or 3 (40%) risk factors (*p* < 0.001). The risk of complications and revision surgery, especially fusion, was also the lowest in patients with ideal indications but steadily increased with the accumulation of risk factors.

**Level of Evidence:**

III.

## Introduction

Anterior vertebral tethering (AVT) is a minimally invasive alternative to fusion surgery for adolescent idiopathic scoliosis (AIS) that offers the potential for definitive scoliosis treatment without sacrificing the growth, motion, or function of the spine. Additionally, the flexible correction of spinal deformity with AVT may also preserve the overall health of the spine by reducing the future risk of adjacent segment degeneration related to rigid instrumentation and fusion over multiple spinal segments [1–7]. Despite significant enthusiasm for AVT, however, the earliest studies of this new treatment have been somewhat disappointing due to a relatively high rate of reported complications (12–48%) and additional procedures (12–41%) [8–16]. Though these early investigations have been useful in providing a better understanding of the challenges of AVT, most represent learning curve data that often provide only the initial experience of a center or group of centers without a substantive critique of surgical indications aimed at improving outcomes.

The earliest published indications for AVT were provided by the FDA in 2019 as part of the first HDE approval of a tether device for AIS. These indications recommended treatment of curves in a 30–65° range in a skeletally immature patient. Perhaps, because these indications were considered too broad to guide the specific treatment of any given patient, a Joint SRS/POSNA Position Statement was subsequently issued in 2020 that modified these indications [17, 18]. Though the SRS and POSNA echoed the FDA curve magnitude recommendations of 30–65°, they narrowed the skeletal maturity indications to Risser ≤ 2 or Sanders ≤ 5. While no specific data were provided to support these particular skeletal maturity indications, the rationale offered for the restricted range was the belief that the definition of skeletal immaturity should be similar to that used for bracing indications. Despite these early efforts to improve the indications for AVT, the guidelines for this novel treatment have remained suboptimal and, in some circumstances, may invite certain complications. For example, the treatment of curves at the lower end of the 30–65° range, in patients at the extreme of skeletal immaturity (Risser –1 or Sanders 1–2), significantly increases the risk of overcorrection, adding-on, and deformity progression after tether rupture. Additionally, the treatment of larger, stiffer curves, at the upper end of the 30–65° range, significantly increases the risk of inadequate correction. Overcorrection, adding-on, deformity progression after tether rupture, and inadequate correction are the most common complications after AVT and are concerning because they often require revision surgery including conversion to fusion [10, 11, 13, 15, 16, 19, 20].

A number of authors have subsequently offered guidelines for AVT treatment that suggest more specific ranges of curve magnitude, skeletal maturity, and even curve flexibility, but these recommendations remain somewhat limited in their usefulness because they do not allow stratification of patients pre-operatively by risk factors or post-operatively by outcomes [11, 19–27]. Additionally, the majority of these recommendations were derived from studies with a relatively narrow population of AIS patients who had a single thoracic curve pattern (e.g., Lenke 1A) treated at the extreme of skeletal immaturity (e.g., Risser –1). To our knowledge, we are the only group that has stratified AIS patients pre-operatively according to multiple risk factors and post-operatively according to outcomes. We are also the only group that has sought to establish indications for AVT using an AIS population with a broad range of curve magnitudes, curve types, and curve flexibilities across the full spectrum of skeletal immaturity to maturity.

The purpose of this study was to analyze a large, consecutive series of AIS patients treated with AVT by a single lead surgeon over a 13 year period with the goal of identifying ideal versus acceptable indications for this novel procedure. Patients were categorized as having ideal indications if they had no risk factors related to curve magnitude, curve flexibility, or skeletal maturity. That is, they were considered to have ideal indications if they had curves in the 40–60° range, curve flexibility ≥ 50%, and skeletal maturity graded at Risser 0–2 and/or Sanders 3–5. Patients with acceptable indications were stratified into three groups depending on the number of risk factors present (1, 2, or 3). Risk factors were assigned to patients if their curve magnitude, curve flexibility, and/or skeletal maturity were out of the ideal range. Our primary hypothesis was that outcomes after AVT would be better in patients with ideal indications and no risk factors. Our secondary hypothesis was that the accumulation of multiple risk factors in patients with acceptable indications would decrease the chance of a good or excellent outcome and increase the chance of complications and revision surgery.

## Methods

Under IRB approved protocols, a retrospective analysis was performed on 185 AIS patients consecutively treated with AVT from 2010 to 2023 with 140 patients achieving a minimum of 2 years follow-up (range 2–11). The range of curve magnitude was 33–72° with skeletal maturity spanning Risser − 1 to 5 (Risser −1 indicating Risser 0 with open triradiate cartilages) and Sanders 2–8. Both single and double curve patterns were treated in the thoracic and thoracolumbar spine spanning Lenke types 1, 2, 3, 4, 5, and 6. Charts and radiographs were reviewed to establish basic demographic data and identify complications and additional procedures. All patients had standard posteroanterior and lateral full length scoliosis radiographs and bending films pre-operatively. The Cobb method was used to measure curve magnitude on pre-operative, post-operative, and final radiographs. Skeletal maturity was assessed using the Risser sign and Sanders stage.

Patients were divided into four groups depending on the number of risk factors they had pre-operatively. The first group had ideal indications and no risk factors. Ideal indications included curve magnitude of 40–60°, curve flexibility of ≥ 50%, and skeletal maturity graded at Risser 0–2 and/or Sanders 3–5. The next three groups had acceptable indications with 1, 2, or 3 risk factors. Those patients considered to have 1, 2, or 3 risk factors failed to meet 1, 2, or 3 of the ideal criteria. Radiographic outcomes were graded as excellent for final curves ≤ 25°, good for final curves 26–39°, fair for final curves ≥ 40°, and poor if the final curve was ≥ 50° or required conversion to fusion.

The surgical technique for this procedure has been described previously by our group [15, 28, 29]. In brief, all patients were placed in a lateral decubitus position for surgery. A 3-portal endoscopic approach with single lung ventilation was used for thoracic curves, allowing access from T4 to L2. A mini-open approach was used for thoracolumbar curves with access possible from T9 to L4. The majority of the diaphragm was preserved in all cases. Segmental vessels were sacrificed prior to bicortical screw placement under fluoroscopic guidance. Most patients with thoracic and thoracolumbar curves were instrumented with a single polyethylene-terephthalate (PET) cord spanning Cobb end vertebra to end vertebra. A small number of more mature patients with thoracolumbar curves were instrumented in more recent years with a double PET cord. The PET cord was differentially tensioned under fluoroscopic guidance to achieve the desired correction of disk angulation at each level. Tensioning proceeded from cephalad to caudad with maximal tension applied to the apical segments and less tension across the end vertebrae. A temporary chest tube was used to assist with reinflation of the lung and was routinely removed, under appropriate circumstances, at the end of the procedure [30]. Spinal cord monitoring was used in all cases. Patients with double curve patterns had both curves treated under one anesthetic with the thoracic curve treated first. An access surgeon was utilized for all procedures.

Hydroxyapatite coated titanium screws and a PET cord from the Dynesys Dynamic Stabilization System (Zimmer Biomet Spine, Broomfield, CO) were used in all cases without the polycarbonate-urethane spacer during the first 10 years of the study. As this device system was approved by the FDA in 2003 for adult lumbar spine stabilization as an adjunct to fusion, its use in the treatment of scoliosis in children during this portion of the study was considered an off-label indication. During the last three years of the study, hydroxyapatite coated titanium screws and PET cords from The Tether System (Highridge, Westminster, CO) were used. This device system achieved FDA approval in 2019.

Standard statistical analysis was performed using paired t-tests, one-sided t-tests, or chi-square analysis, where appropriate, to determine the significance of comparisons between two or more groups with two or more variables. Importantly, though this study identified four groups (ideal, 1 risk factor, 2 risk factors, 3 risk factors) and four outcomes (excellent, good, fair, poor), a simplified analysis using t-tests was employed after combining the groups (ideal and 1 risk factor versus 2 and 3 risk factors) and the outcomes (excellent and good versus fair and poor). Additionally, a 2 × 2 contingency table was used to calculate and examine the association between each individual risk factor and the odds of a good or excellent outcome. Odds ratios (OR) and their corresponding 95% confidence intervals (CI) were calculated. All statistical analyses were conducted in Microsoft Excel for Mac (version 16.52; Microsoft) with an alpha set at 0.05.

## Results

**Fig. 1:**
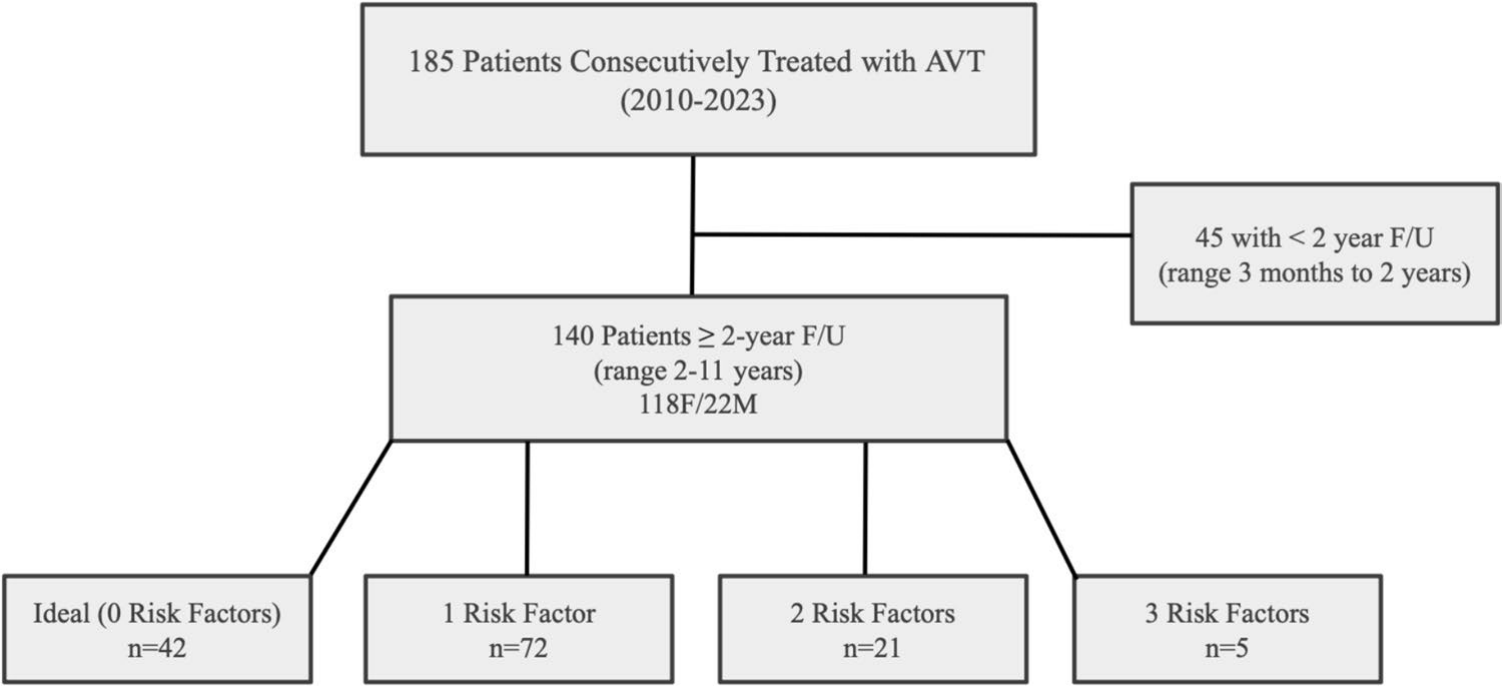
Of 185 AIS patients consecutively treated with AVT over 13 years, 140 with ≥ 2 year follow-up were divided into four groups depending on the number of risk factors present

**Fig. 2:**
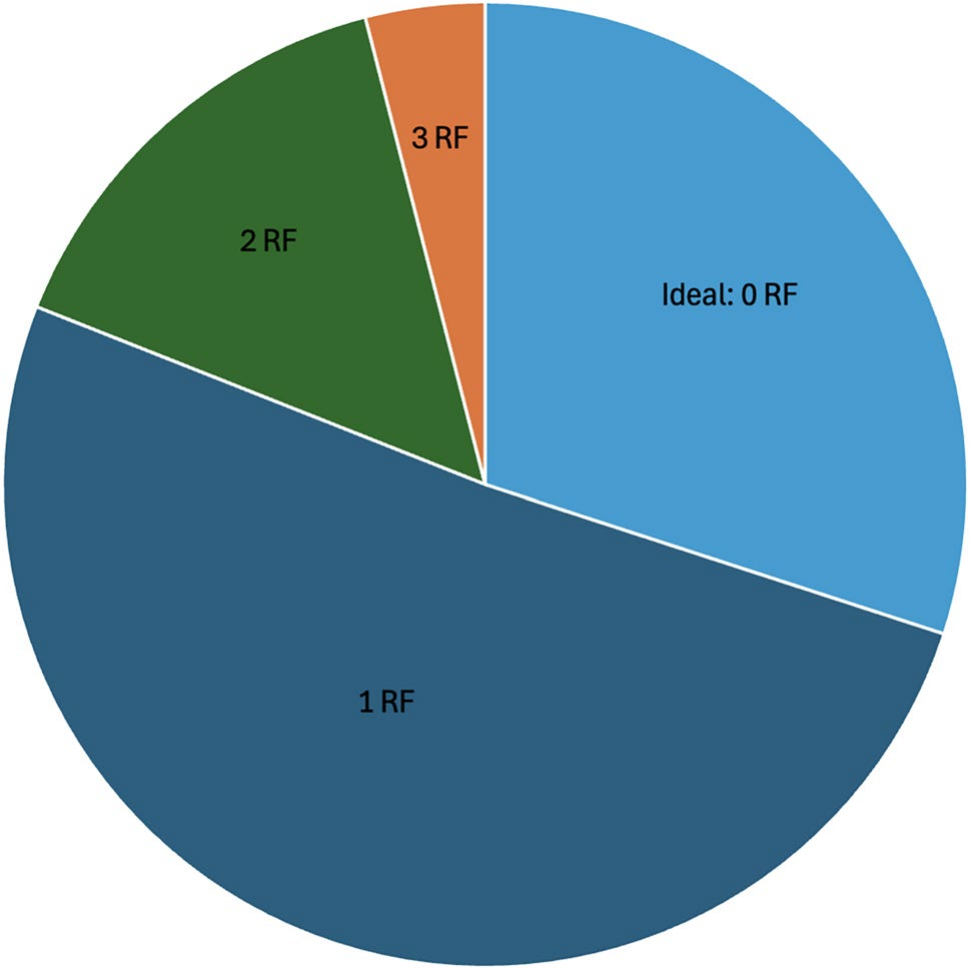
Of 140 AVT patients treated over a 13 year period with 2-year follow up, 30% had ideal indications with no risk factors, 51% had acceptable indications with 1 risk factor, 15% had acceptable indications with 2 risk factors, and 4% had 3 risk factors

**Fig. 3:**
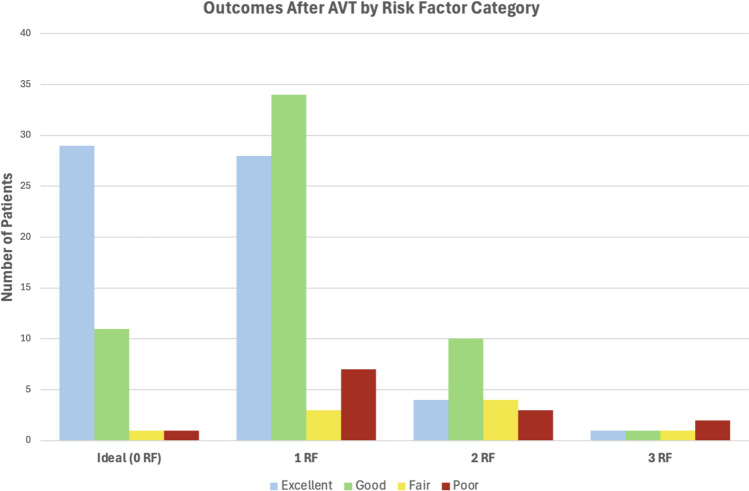
Outcomes in 140 AIS patients treated with AVT by risk category. Patients with ideal indications and no risk factors had a 95% chance of a good or excellent outcome (71% excellent/24% good); patients with acceptable indications and 1 risk factor had an 86% chance of a good or excellent outcome (46% excellent/40% good); patients with acceptable indications and 2 risk factors had a 67% chance of a good or excellent outcome (19% excellent/48% good); and patients with 3 risk factors had only a 40% chance of a good or excellent outcome (20% excellent/20% good)

One hundred and forty patients (118F/22 M) with 199 curves (59 thoracic, 22 thoracolumbar, 59 double curves) had 3.2 years (range 2–11) follow-up after treatment with AVT for pre-operative curves of 49.8° at 14.1 years of age and skeletal maturity of Risser 2.4 and Sanders 5.0 (Fig. 1 and Table 1). Thirty percent of patients treated (42/140) had ideal indications and no risk factors, whereas 51% (72/140) had acceptable indications and 1 risk factor, 15% (21/140) had acceptable indications and 2 risk factors, and 4% (5/140) had 3 risk factors (Fig. 2). The proportion of patients with good or excellent outcomes at a final follow-up of ≥ 2 years was highest in the patients with ideal indications and no risk factors but decreased steadily with the accumulation of 1, 2, or 3 risk factors (Fig. 3). Though a trend toward a significantly decreased chance of a good or excellent outcome was evident between all groups as they accumulated risk factors, this only achieved significance when ideal patients were compared to patients with 2 or 3 risk factors or when patients with 1 risk factor were compared to those with 3 risk factors (Table 2). Additionally, a combined group of patients with ≤ 1 risk factor, including those with ideal indications and no risk factors, demonstrated a significantly greater chance of a good or excellent outcome when compared to a combined group of patients with ≥ 2 risk factors (*p* < 0.001). Importantly, the majority of patients with 0, 1, and even 2 risk factors had good or excellent outcomes. However, the majority of patients with 3 risk factors had fair or poor outcomes rather than good or excellent outcomes.

**Table 1:**
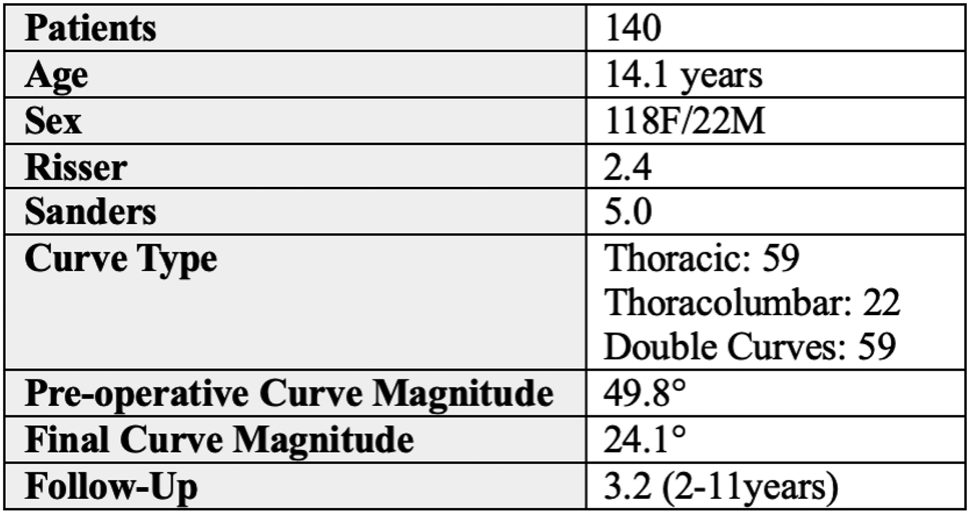
Demographic and radiographic data for 140 AIS patients with ≥ 2 year follow-up stratified by curve location and the number of curves treated

**Table 2:**
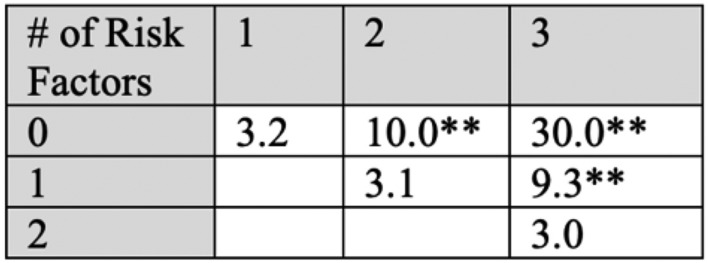
Odds ratios were calculated between each of the four groups with significance demonstrated between the ideal patient group (0 risk factors) and those with 2 or 3 risk factors as well as between the 1 risk factor group and those with 3 risk factors

**Fig. 4:**
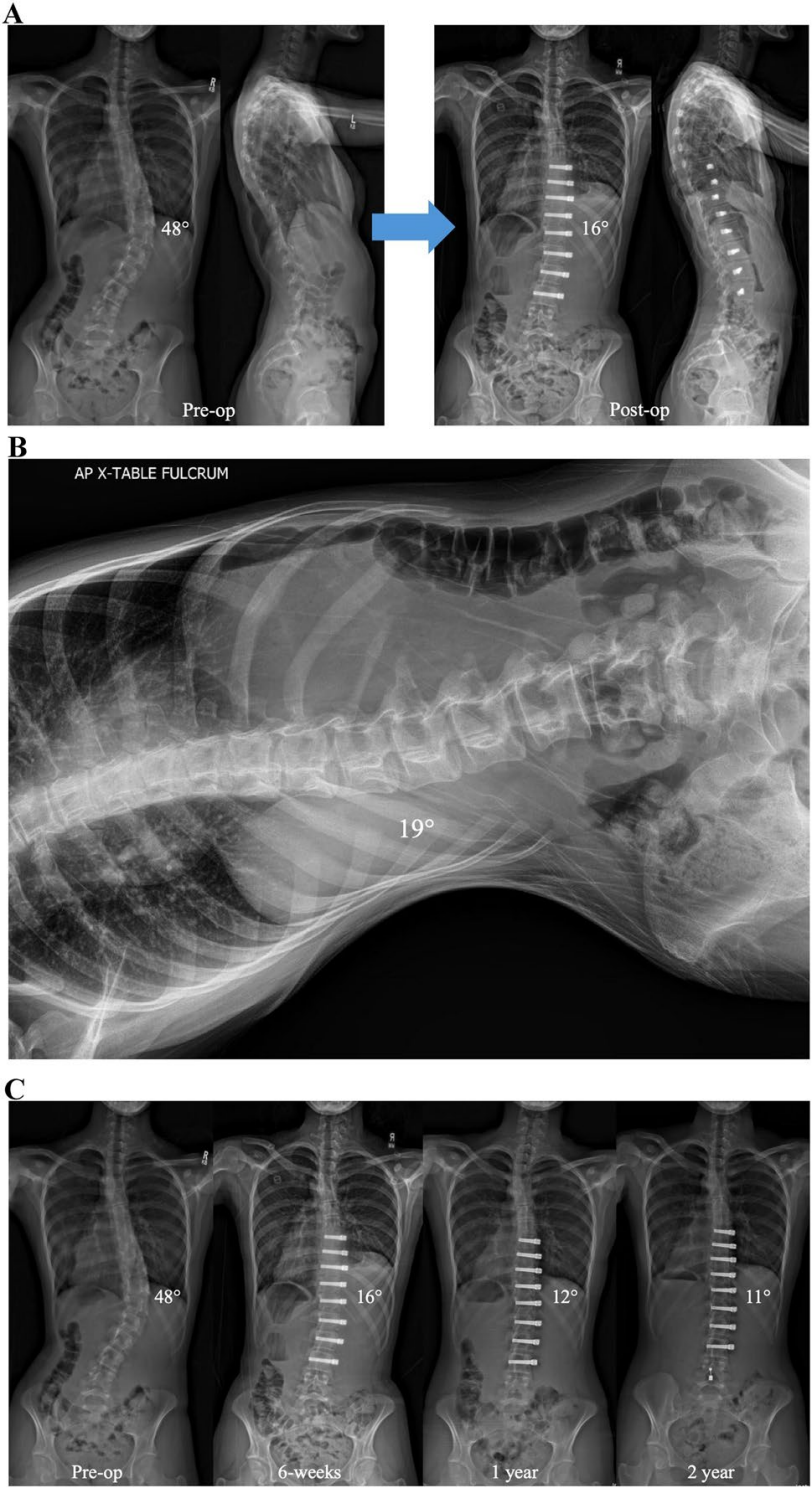
**A** Pre-operative and post-operative posteroanterior and lateral radiographs of an AIS patient with ideal indications for AVT: a 14 + 3 year-old female with a Lenke 5C curve pattern involving a 48° curve right T8-L3 at Risser 0 and Sanders 6 with 60% curve flexibility. AVT right T8-L3 resulted in initial curve correction from 48 to 16° **B** Fulcrum bending film demonstrates 60% flexibility of the thoracolumbar curve, bending from 48 to 19°.**C** Pre-operative, post-operative, 1 year, and 2 year posteroanterior radiographs demonstrating an excellent outcome with initial curve correction from 48 to 16° and additional growth modulation to 12° and 11° at 1 and 2 years, respectively

Patients with ideal indications who had no risk factors (*n* = 42) demonstrated 95% good or excellent outcomes (40/42) with curve correction from 48.3 pre-operatively to 21.5° post-operatively to 19.6° final at 2.6 years (Fig. 4). These good or excellent outcomes were achieved despite a 26% tether rupture rate but with no requirement for revision surgery. In the 5% of patients with fair or poor outcomes (2/42), curves corrected from 52.0 pre-operatively to 30.3° post-operatively to 41.7° final at 2.0 years. In one patient with a fair outcome despite ideal indications, a single thoracic curve that corrected from 52 to 38° after AVT lost correction due to proximal adding-on coincident with subtle migration of the T6 vertebral screw in the setting of poor bone quality. This patient’s final curve magnitude of 42° remains asymptomatic and has not required revision surgery to date. In the other patient with a poor outcome despite ideal indications, thoracic curve correction from 57 to 35° and lumbar curve correction from 44 to 23° after AVT was lost post-operatively due to early lumbar tether ruptures at L1,2 and L2,3 at one year. Revision surgery involving fusion of the 40° thoracic curve alone, without the need for revision of the 32° lumbar curve, was eventually required in this patient with final curves of 12° and 15° degrees, respectively [16].

**Fig. 5:**
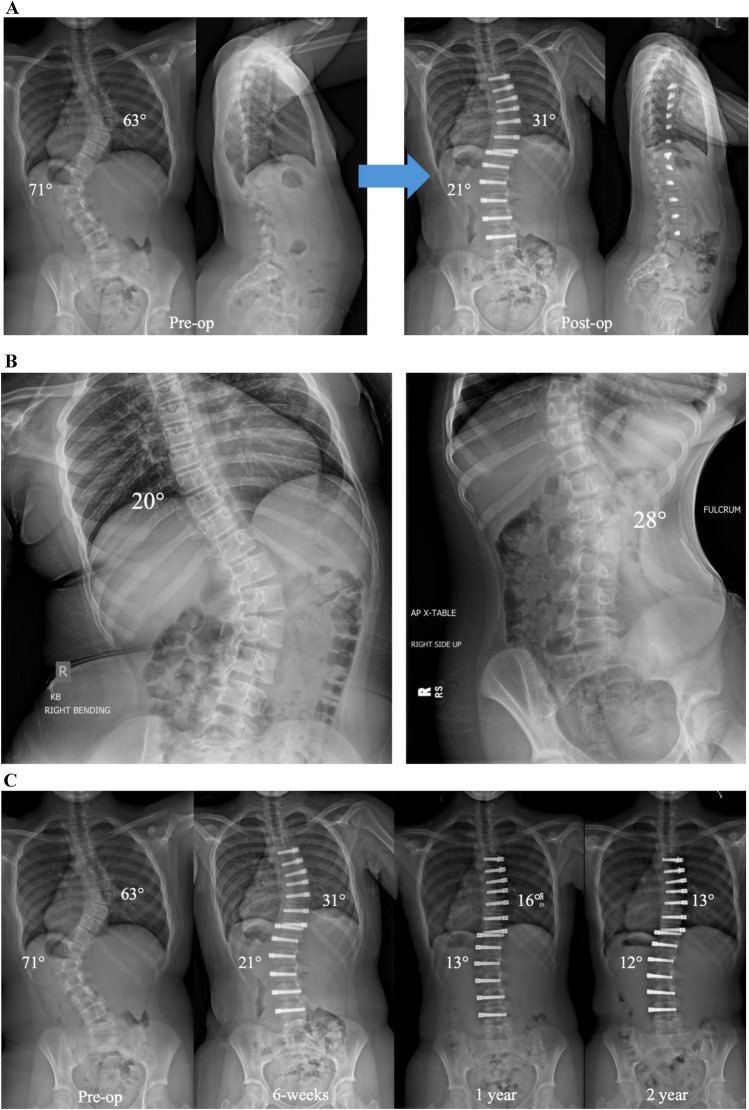
**A** Pre-operative and post-operative posteroanterior and lateral radiographs of an AIS patient with acceptable indications for AVT but 1 risk factor (curve magnitude): 11 + 4 year-old female with a Lenke 6C curve pattern involving a 63° curve right T5-T11 and 71° curve left T11-L3 at Risser 0 and Sanders 3 with 68% thoracic curve flexibility and 61% thoracolumbar curve flexibility. AVT right T5-T11 and left T11-L3 resulted in initial thoracic curve correction from 63 to 31° and thoracolumbar curve correction from 71 to 21°. Please note this patient has 11 thoracic rib bearing segments with no ribs evident at T12. **B** A standard right thoracic bending film demonstrated 68% flexibility of the thoracic curve, bending from 61 to 20°. The thoracic fulcrum bending film demonstrated less flexibility and therefore is not shown. A fulcrum bending film of the left thoracolumbar curve demonstrated 61% flexibility, bending from 71 to 28° **C** Pre-operative, post-operative, 1 year, and 2 year posteroanterior radiographs demonstrating an excellent outcome after initial curve corrections from 63 to 31° and 71 to 21° with additional growth modulation of both curves to 13° and 12°, respectively, at 2 years

Patients with acceptable indications who had 1 risk factor (*n* = 72) demonstrated 86% good or excellent outcomes (62/72) with curve correction in these patients from 48.5 pre-operatively to 23.0° post-operatively to 21.9° final at 3.0 years (Fig. 5). These good or excellent outcomes were achieved with a 33% tether rupture rate but no requirement for revision surgery. In the 14% of patients with 1 risk factor and fair or poor outcomes (10/72), curves corrected from 51.9 pre-operatively to 34.7° post-operatively to 43.2° final at 3.5 years. In these 10 patients there were five with tether rupture, four with inadequate correction, and one with overcorrection. In the five patients with tether rupture, four required revision surgery. Two of these patients required thoracic fusion alone, two required tether revision alone, and one currently has an asymptomatic 40° thoracic curve that has not required revision surgery to date. In the four patients with inadequate curve correction, all four required revision surgery involving conversion to fusion. The single patient with overcorrection also required conversion to fusion.

**Fig. 6:**
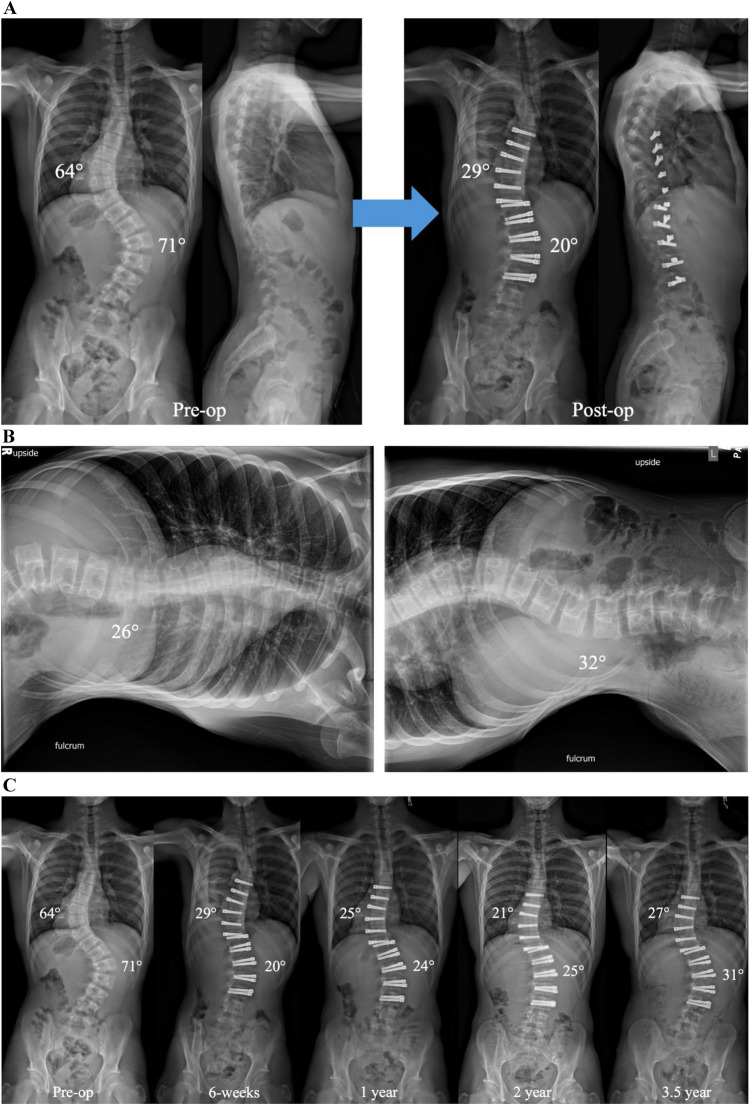
**A** Pre-operative and post-operative posteroanterior and lateral radiographs of an AIS patient with acceptable indications for AVT but 2 risk factors (curve magnitude and skeletal maturity): 14 + 10 year-old male with a Lenke 6C curve pattern involving a 64° curve left T7-T12 and 71° curve right T12-L4 at Risser 3 and Sanders 5 with 59% thoracic curve flexibility and 54% thoracolumbar curve flexibility. AVT left T7-T12 and right T12-L4 with a double cord resulted in initial thoracic curve correction from 64 to 29° and thoracolumbar curve correction from 71 to 20°. Please note that this patient has six non rib bearing lumbar segments **B** A left thoracic fulcrum bending film demonstrated 59% flexibility of the thoracic curve, bending from 64 to 26°. A fulcrum bending film of the right thoracolumbar curve demonstrated 54% flexibility, bending from 71 to 32° **C** Pre-operative, post-operative, 1 year, 2 year, and 3.5 year posteroanterior radiographs demonstrating a good outcome after initial curve corrections from 64 to 29° and 71 to 20° with some settling of both curves overtime to 27° and 31°, respectively, at 3.5 years possibly related to tether ruptures at T9,10, L2,3, and L3,4

Patients with acceptable indications who had 2 risk factors (*n* = 21) demonstrated 67% good or excellent outcomes (14/21) with curve correction in these patients from 50.8 pre-operatively to 27.1° post-operatively to 24.8° final (Fig. 6). These good or excellent outcomes were achieved with a 29% tether rupture rate with the requirement for revision surgery in 7%. In the 33% of patients with 2 risk factors and fair or poor outcomes (7/21), curves corrected from 52.0 pre-operatively to 27.7° post-operatively to 43.3° final at 2.0 years. In these seven patients there were three tether ruptures, three inadequate corrections, and one overcorrection. Conversion to fusion in this group was required for one of the three inadequate corrections and for the one overcorrection.

**Fig. 7:**
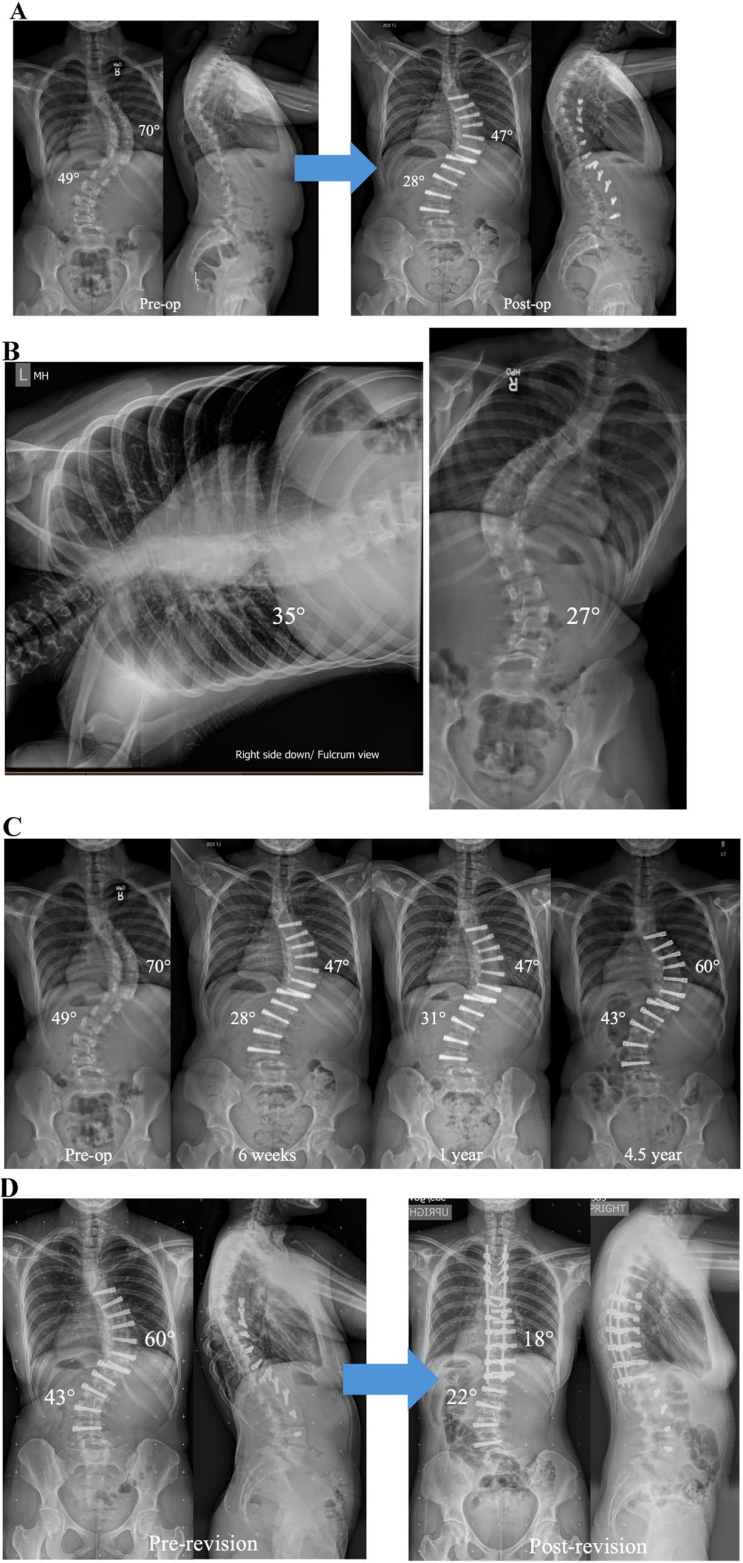
**A** Pre-operative and post-operative posteroanterior and lateral radiographs of an AIS patient with 3 risk factors (curve magnitude, curve flexibility, and skeletal maturity): 14 + 1 year-old female with a Lenke 1B curve pattern involving a 70° curve right T6-T12 and 49° curve left T12-L4 at Risser 4 and Sanders 6 with 50% thoracic curve flexibility and 45% thoracolumbar curve flexibility. AVT right T6-T12 and left T12-L4 resulted in initial thoracic curve correction from 70 to 47° and thoracolumbar curve correction from 49 to 28° **B** A thoracic fulcrum bending film demonstrated 50% flexibility of the thoracic curve, bending from 70 to 35°. A lumbar bending film demonstrated 45% flexibility of the thoracolumbar curve, bending from 49 to 27°. A lumbar fulcrum bending film could not be obtained in this patient.** C** Pre-operative, post-operative, 1 year, and 4.5 years posteroanterior radiographs demonstrating a poor outcome after initial curve corrections from 70 to 47° and 49° to 28° with a slow loss of curve correction in both curves overtime to 60° and 43°, respectively, at 4.5 years. This patient’s poor outcome is likely related not only to the initial inadequate correction of the thoracic curve, but also loss of correction in both curves over time due to multiple tether ruptures (T11,12, L2,3, and L3,4) **D** Pre-operative and post-operative posteroanterior and lateral radiographs before and after revision surgery at 4.5 years involved posterior spinal fusion with pedicle screw instrumentation T3-T12 without the need for revision of the lumbar tether. Revision surgery in this patient resulted in a hybrid construct that limited the fusion to the thoracic spine without any sacrifice of lumbar motion segments. Instrumented spinal fusion allowed direct correction of the thoracic curve from 60 to 18° and indirect correction of the lumbar curve from 43 to 22°. While preservation of lumbar motion segments is an important goal in most AIS patients, it may have an additional benefit in this particular patient by avoiding any concentration of forces in the lower lumbar spine where a grade 2 L5 spondylolisthesis exists

Patients who had 3 risk factors (*n* = 5) demonstrated 40% good or excellent outcomes (2/5) with curve correction from 59.8 pre-operatively to 30.0° post-operatively to 23.8° final at 3.0 years. These good or excellent outcomes were achieved with a 0% tether rupture rate. In the 60% of patients with 3 risk factors and fair or poor outcomes (3/5), curves corrected from 63.6 pre-operatively to 43.8° post-operatively to 50.4° final at 3.0 years. In these three patients with 3 risk factors and fair or poor outcomes, all three had inadequate corrections and one has already undergone conversion to fusion (Fig. 7).

## Discussion

In this study of 140 AIS patients treated with AVT over a 13 year period, outcomes were better in those patients with ideal indications versus those with 1, 2, or 3 risk factors. Patients with ideal indications (curve magnitude 40–60°, curve flexibility ≥ 50%, and skeletal maturity graded at Risser 0–2 and/or Sanders 3–5) had the best chance of achieving an excellent outcome at 71% with an additional 24% of patients in this group achieving a good outcome for a total of 95% of ideal patients achieving good or excellent outcomes. Patients with acceptable indications and 1 risk factor were more likely to achieve a good outcome at 46% but still had a 40% chance of an excellent outcome for a total of 86% achieving good or excellent outcomes. Patients with acceptable indications and 2 risk factors were also more likely to achieve a good outcome at 48% with a lower chance of an excellent outcome at 19% for a total of 67% good or excellent outcomes.

And, finally, patients with 3 risk factors were the least likely to achieve good or excellent outcomes with 20% good and 20% excellent outcomes for a total of 40% good or excellent outcomes.

Though there were several significant differences demonstrated between these four groups of patients, perhaps, the most meaningful analysis involved a comparison of combined groups. That is, a comparison of the chance for a good or excellent outcome in patients with ≤ 1 risk factor (ideal patients and patients with 1 risk factor) versus patients with ≥ 2 risk factors (patients with 2 and 3 risk factors). This comparison yielded a threshold at ≤ 1 risk factor that identified the most attractive candidates for AVT with the greatest chance of a good or excellent outcome (*p* < 0.001).

**Fig. 8:**
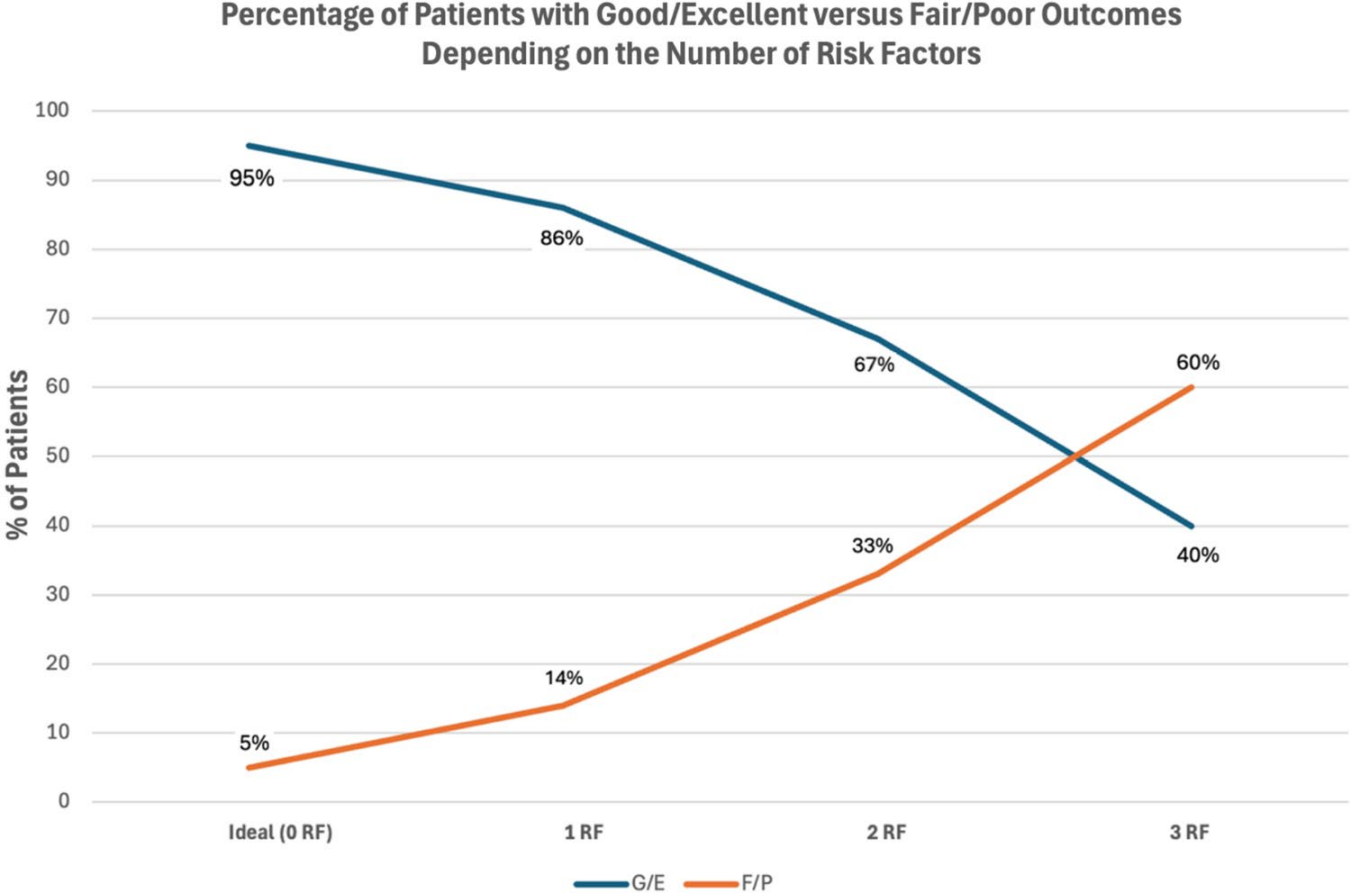
The percentage of patients with a good or excellent outcome is contrasted with the percentage of patients with a fair or poor outcome for each risk category. While patients with 1 or 2 risk factors did demonstrate a decreased chance of a good/excellent outcome, compared to patients with ideal indications, the accumulation of 3 risk factors actually inverted the risk profile. That is, patients with 3 risk factors had a greater chance of a fair/poor outcome rather than a good/excellent outcome

Viewed from the opposite perspective, ideal patients with no risk factors had the lowest chance of a fair or poor outcome with 2.5% fair and 2.5% poor outcomes for a total of 5% achieving fair or poor outcomes. Only one of the two patients with no risk factors and a fair or poor outcome had revision surgery requiring conversion to fusion. Patients with acceptable indications and 1 risk factor had a 4% chance of a fair outcome and a 10% chance of a poor outcome for a total of 14% achieving a fair or poor outcome. Nine of the 10 patients with 1 risk factor and a fair or poor outcome had revision surgery with seven requiring conversion to fusion. Patients with acceptable indications and 2 risk factors had a 19% chance of a fair outcome and a 14% chance of a poor outcome for a total of 33% achieving fair or poor outcomes. Two of the seven patients with 2 risk factors and a fair or poor outcome required revision surgery with both requiring conversion to fusion. Patients with 3 risk factors had the highest chance of a fair or poor outcome with 20% fair and 40% poor outcomes for a total of 60% achieving fair or poor outcomes. One of the three patients with 3 risk factors and a fair or poor outcome has already undergone revision surgery requiring conversion to fusion. Because of the high likelihood of a fair or poor outcome in patients with 3 risk factors, it would not be unreasonable to recategorize this group as having an unacceptable rather than acceptable number of risk factors (Fig. 8).

With respect to the specific type of risk factor and its potential impact on outcomes, an analysis of patients with a single risk factor revealed that skeletal maturity, out of the ideal range of Risser 0–2 and/or Sanders 3–5, was the most common single risk factor. Though this single risk factor was present in 67% (48/72) of patients in this group, 94% (45/48) still achieved a good or excellent outcome. Importantly, all patients with this single risk factor who achieved a good or excellent outcome had greater skeletal maturity (Risser 3–5 and/or Sanders 6–8) than the ideal patients, rather than excessive immaturity (Risser − 1 and/or Sanders 1–2). Patients with the single risk factor of reduced curve flexibility had less of a chance of a good or excellent outcome at 80% (8/10) while those with the single risk factor of increased curve magnitude had the least chance of a good or excellent outcome at 64% (9/14). A comparison of these 3 risk factors demonstrated a significantly greater chance of a good or excellent outcome with skeletal maturity as a single risk factor versus increased curve magnitude (OR = 8.52, 95% CI = 1.72–42.18, *p* < 0.05). And though there was a trend toward significance when comparing the chance of a good or excellent outcome with skeletal maturity versus curve flexibility or curve flexibility versus curve magnitude as single risk factors, neither achieved significance. In sum, greater skeletal maturity was not only the most common risk factor but also the best tolerated single risk factor in this study, when compared to curve magnitude or curve flexibility, and was associated with the highest likelihood of a good or excellent outcome at 94%.

For patients with acceptable indications and 2 risk factors, our initial expectation was that the combination of increased curve magnitude and reduced curve flexibility would be the most problematic for patients after AVT. However, all three combinations of two risk factors (skeletal maturity and curve magnitude; skeletal maturity and curve flexibility; and curve magnitude and curve flexibility) yielded approximately the same risk of a fair or poor outcome. Those patients with combined risk factors related to both skeletal maturity and curve magnitude had a 33% (2/6) chance of a fair or poor outcome. This was similar to those with combined risk factors related to skeletal maturity and curve flexibility (8/12) and curve magnitude and curve flexibility (2/3), each also demonstrating a 33% chance of a fair or poor outcome. Though only present in one patient, the one dual risk factor type that did seem to be highly associated with a poor outcome was the combination of reduced curve magnitude (< 40°) and excessive immaturity (Risser −1), resulting in significant overcorrection and eventual conversion to fusion.

Though the aim of this study was not to evaluate the impact of curve type on outcomes after AVT, the distribution of curve types among all patients treated was found to be weighted toward thoracic dominant curve patterns (61% Lenke 1, 2% Lenke 2, 1% Lenke 4) versus other curve types (7% Lenke 3, 11% Lenke 5, and 18% Lenke 6). Among fair or poor outcome patients, an even greater prevalence of thoracic dominant curve patterns was noted at 82% (73% Lenke 1, 9% Lenke 2, and 0% Lenke 4) versus 64% overall. Among fair or poor outcome patients, a decreased prevalence of lumbar dominant curve patterns was also noted at 14% (0% Lenke 5 and 14% Lenke 6) versus 29% overall. Additionally, an absence of double major curves was noted in patients with fair or poor outcomes (0% Lenke 3) versus 7% overall.

While a direct comparison of our results to other published series may be difficult– as no other study has attempted to stratify AVT patients pre-operatively by risk category or post-operatively by outcome– we analyzed our outcomes, for reference, in a dichotomous manner similar to that used in other studies. Many of these studies use a single curve correction value as a threshold for success versus failure after AVT. In one of the earliest reports on tether surgery, for example, Newton chose 35° as a threshold for success versus failure and found 58% of patients in his study achieved a curve correction of < 35° [10]. Rushton chose a similar 35° threshold for success and found 71% of patients in their study achieved a final curve correction of < 35° [14]. Hoernschemeyer and Samdani both chose 30° as a threshold for success with 74% and 80% of patients in their two studies achieving ≤ 30° and < 30° of correction, respectively [11, 13]. Though our overall data, including all patients treated over the 13 year period, compares favorably to these published studies– with 61% of our patients achieving a curve correction ≤ 30° and 75% ≤ 35°– further stratification not only by outcome but also by risk category provides a better understanding of the impact of these ideal indications on the degree of success after AVT. In our ideal patient group, representing 30% of patients treated, we found 63% ≤ 25°, 80% ≤ 30°, 88% ≤ 35°, and 95% ≤ 39°. In a larger combined group, representing 81% of patients treated (30% with ideal indications and 51% with acceptable indications and 1 risk factor), we found 48% ≤ 25°, 66% ≤ 30°, 80% ≤ 35°, and 91% ≤ 39°.

The primary limitations of this study are related to its retrospective nature and the absence of a patient reported outcomes measure. Without patient reported measures, it could be argued that the radiographic outcomes presented in this study do not represent the full clinical picture for this patient cohort. While this may be true, the study does represent a first attempt to stratify AIS patients pre-operatively by risk factors in order to predict radiographic success after AVT– with the thought that radiographic success after AVT likely contributes significantly to the achievement of overall clinical success.

Additional limitations encountered in this study include a substantial evolution in the indications for treatment and for revision surgery over the 13 year study period. Though our indications for the treatment of AIS patients with this new procedure were not unreasonable at the outset, these indications, certainly, improved over time with experience. For example, in our earliest years, spanning 2010–2012, it was not clear that AVT would be effective in the treatment of curves > 40° and, therefore, we initially targeted curves in the 35–40° range [15, 28]. However, due to the success of the tether in correcting scoliosis in these smaller curves, we learned that we could effectively treat larger curves in the 40–60° range. During these early years we also learned to avoid the treatment of smaller curves < 40° in extremely skeletally immature patients (Risser − 1) as these proved to be at significantly increased risk of overcorrection.

By 2012 we had, essentially, established a lower end of the ideal threshold for AVT treatment with a curve magnitude of 40° and skeletal maturity at Risser 0. Eventually, we also learned that we could successfully treat more flexible curves > 60°. However, it was not clear for some time how much flexibility would be necessary to achieve a satisfactory result. And, additionally, during these early years, it became apparent that the significant initial correction of curves at the time of surgery could allow for the effective management of flexible curves with AVT in more mature adolescents even without the potential for growth modulation.

Because the risk profile for AVT was poorly understood during these earliest years, the timely identification and optimal management of complications also proved challenging. Unfortunately, this resulted in a higher rate of revision surgery for tether rupture and a higher rate of conversion to fusion for overcorrection in the early versus later years (15). With an evolution in our understanding of tether rupture, including evidence that most tether ruptures are inconsequential, we reduced our rate of revision surgery for tether rupture over time (16). Similarly, an evolution in our understanding of overcorrection has nearly eliminated this complication in our patient cohort over time. However, when this complication is encountered, early intervention has allowed for definitive treatment with tether removal alone rather than requiring conversion to fusion.

All of the above observations ultimately led to our selection of what we consider to be the three most important factors in indicating AIS patients for AVT. Though the ideal curve magnitude, curve flexibility, and skeletal maturity indications we have offered will likely benefit from additional refinement—perhaps, by grading the severity of each risk factor, by improving our understanding of the relationship between risk factors, and even by inclusion of additional risk factors—the simplicity of these current indications provides a robust guide for the treatment of AIS patients with AVT with a high chance of success.

## Data Availability

None applicable.
